# A Descriptive Study Examining Trends in Pharmacist-Authored Original Research Publications in the *Journal of the American Medical Association Network* from 2000 to 2019

**DOI:** 10.3390/pharmacy9010040

**Published:** 2021-02-13

**Authors:** Delaney M. Strong, Kevin T. Fuji

**Affiliations:** 1 School of Pharmacy and Health Professions, Creighton University, Omaha, NE 68178, USA; 2 Department of Pharmacy Practice, School of Pharmacy and Health Professions, Creighton University, Omaha, NE 68178, USA; KevinFuji@creighton.edu

**Keywords:** authorship, pharmacy research, research training, medical journals, bibliometrics, publishing trends

## Abstract

Pharmacists are expected to participate in the conduction of research to advance the profession and health care broadly. Additional opportunities for pharmacist research engagement have emerged with the increased integration of clinically trained pharmacists into interprofessional care teams. Research conducted over the past four decades has demonstrated an increasing trend of pharmacist-authored publications in medical journals. The purpose of this study was to build upon this work and investigate trends in pharmacist-authored original research publications within the *JAMA Network* over the past 20 years. A descriptive study design was used to retrospectively evaluate trends in the numbers of pharmacist-authored publications and authorship within those publications in nine *JAMA Network* journals. Data were aggregated into ten-year time periods (2000–2009 and 2010–2019) and compared using chi-square and Fisher’s exact tests. Overall, pharmacist-authored publications significantly increased over the ten-year period (2.0% to 3.0%, *p* < 0.001), including in five specific journals: *JAMA*, *JAMA Dermatology*, *JAMA Neurology*, *JAMA Ophthalmology*, and *JAMA Surgery*. There was no change in first—and senior-authored publications. While the overall pharmacist publication trend was positive, room for significant growth remains. A deeper understanding of the barriers and facilitators to pharmacist engagement in research is needed, along with strategies to enhance pharmacist research training.

## 1. Introduction

Engaging in research and contributing to the peer-reviewed literature base is recognized as an important component of clinical pharmacy training, postgraduate education, and practice; numerous pharmacy organizations in the United States (U.S.) and internationally explicitly identify research as a core aspect of their mission or vision statements and provide resources to support the research efforts of their members [1,2,3,4,5,6,7,8].

Despite the recognition of the importance of research not only to the profession, but to health care broadly, historically, pharmacists’ contributions to the peer-reviewed literature in non-pharmacy-focused health care journals have been limited. Studies published from 1979 to 2008 all noted an increase in pharmacist-authored research publications when examining ten-year intervals, however the rate of pharmacist authorship in major medical journals remains low overall [9,10,11].

Numerous factors could be leading to increases in pharmacist contribution to health care literature in major medical journals in the past decade plus, including overall workforce growth globally and an increasing number of pharmacy colleges and schools in countries such as the United States (U.S.) and United Kingdom, which has led to increases in both pharmacy faculty who have an expectation of research productivity as part of their position and in pharmacy graduates who have the potential to engage in original research studies and subsequent publication [12,13]. Further, as the expertise of pharmacists as medication use experts has increasingly been recognized by other health professions and led to integration of pharmacists within interprofessional health care teams, more opportunities have theoretically been provided for pharmacists to engage in research with other health care professionals [14,15,16,17,18].

As there are no recent publications examining pharmacist publication patterns and how pharmacist involvement in major medical journals has changed over time, this study was conducted to better understand the longitudinal contribution of pharmacists to the health care literature using the *Journal of the American Medical Association* (*JAMA*) Network journals. The *JAMA Network* was chosen due to its recognition as a premier international health care research outlet with high readership, high impact factors, and publication of a variety of article topics, including clinical research, basic science, health policy, and others from both general medical and specialty perspectives. The purpose of this study was to investigate trends in pharmacist-authored publications within the *JAMA Network* over the past 20 years.

## 2. Materials and Methods

A descriptive study design was used to retrospectively assess the prevalence of pharmacist-authored publications across journals in the *JAMA Network* from 2000 to 2019. In order to generate a large enough sample size to support statistical comparison, only journals that had been published for at least 20 years were included in this study. This led to the exclusion of three journals: *JAMA Cardiology* (published since 2016), *JAMA Oncology* (published since 2015), and *JAMA Network Open* (published since 2018). The included journals were: *JAMA*, *JAMA Dermatology*, *JAMA Internal Medicine*, *JAMA Neurology*, *JAMA Ophthalmology*, *JAMA Otolaryngology—Head and Neck Surgery*, *JAMA Pediatrics*, *JAMA Psychiatry*, and *JAMA Surgery*.

For each included journal, the advanced search function on the *JAMA Network* website was used to identify pharmacist-authored publications. For each year from 2000 to 2019, each individual journal was selected; the “Research” article type was selected; and the keywords “PharmD”, “MPharm”, “BPharm”, and “RPh” were used. Search results were screened to exclude all review articles and case studies. Pharmacist-authored publications were further categorized as either a first- or senior-authored publication or a co-authored publication.

To minimize the impact of year-to-year variation, including publication increases related to health innovations and novel disease states, and to support a sufficient sample size within each journal to perform statistical comparisons, the data were aggregated into 10-year periods of 2000–2009 and 2010–2019. The total numbers of pharmacist-authored publications comparing the two 10-year periods were analyzed using a chi-square test for all journals combined, *JAMA*, and *JAMA Internal Medicine*, and a Fisher’s exact test for all other journals, with a significance level set at *p* ≤ 0.05. A sub-analysis of only pharmacist-authored publications was conducted to examine the change in first—or senior-authored articles comparing the same two 10-year time periods, also using a Fisher’s exact test.

## 3. Results

Overall, there was a minimal number of publications with pharmacist authors. From 2000 to 2009, there was a total of 315 (2.0%) pharmacist-authored publications across all journals, accounting for 0.2% to 5.5% of publications within individual journals. From 2010 to 2019, there was a total of 434 (3.0%) pharmacist-authored publications across all journals, accounting for 0.6% to 6.4% of publications within individual journals. The greatest numbers of pharmacist-authored publications in both 10-year periods were found in *JAMA Internal Medicine* and *JAMA*.

The increase in the total number of pharmacist-authored publications from 2000–2009 to 2010–2019 was statistically significant (*p* < 0.001). Although all individual journals showed increases in the percentage of pharmacist-authored publications from 2000–2009 to 2010–2019, only five were statistically significant increases: *JAMA* (3.5% to 4.9%, *p* = 0.020), *JAMA Dermatology* (0.7% to 2.3%, *p* < 0.05), *JAMA Neurology* (0.9% to 2.2%, *p* < 0.05), *JAMA Ophthalmology* (0.7% to 1.6%, *p* ≤ 0.05), and *JAMA Surgery* (0.8% to 1.7%, *p* < 0.05). Table 1 displays the breakdown of pharmacist-authored publications and total research publications for each *JAMA* journal over the aggregated 10-year time periods, while Table 2 displays a year-by-year breakdown of pharmacist-authored publications for each journal.

The sub-analysis of only pharmacist-authored publications to examine changes in first—and senior-authored designations revealed no statistically significant change for any of the journals (see Table 3). Combining data across all journals revealed a decrease in first—and senior-authored publications from 28.0% in 2000–2009 to 26.8% in 2010–2019, which was not a statistically significant difference.

## 4. Discussion

The findings from this study demonstrate that pharmacist-authored publications in *JAMA Network* journals continue to increase over time, similar to prior studies examining earlier time periods and other medical journals [9,10,11]. However, from a practical standpoint, pharmacist-authored publications still account for a small percentage of the total number of publications both within and across *JAMA Network* journals, indicating significant potential for growth. While the reasons for this continued increase, along with associated barriers and facilitators to publication were not explored in this study, there are strategies described in the existing literature that can be employed to both sustain this narrow increase and support future growth.

There is a need to ensure that adequate research training is being provided in the pharmacy curriculum and post-graduate training [11]. Globally, multiple countries have recognized gaps in research training within their pharmacy curricula that can be further exacerbated in post-graduate training, which often lacks formal research training and research mentorship [19,20,21,22,23,24]. Rigorous research training during both professional pharmacy training and post-graduate experiences can lead not only to future publication success, but can also instill a culture of research and inquiry into future practice (regardless of setting) and facilitate opportunities to collaborate with others [25,26,27]. Lack of research instruction for pharmacists whose training primarily focuses on clinical aspects may also be a driver behind junior staff feeling ill-equipped to meet the research expectations of their position, having a smaller number of staff across the academy accounting for a large volume of publications, and having a lower conference abstract to full publication rate compared to other health professions [28,29,30,31,32].

Interprofessional collaboration has been found to facilitate pharmacist publishing, further supported by research demonstrating an increase in physician co-authored publications in prominent pharmacy journals over a 20-year period [14,33]. Just as accreditation standards emphasize student training in interprofessional care, there should also be an emphasis on interprofessional engagement in other areas, such as research [34,35].

Future research should focus on a deeper examination of barriers and facilitators to pharmacist participation in research [14]. Given that many pharmacist publication rate studies were conducted over a decade ago, there is a need to continually update these studies across a wider range of medical and health care journals.

### Limitations

Only articles published in the *JAMA Network* were included in this study. While the *JAMA Network* journals are international publications with high readership and a focus on original research in general medicine and specialty areas, pharmacists publish in a wide range of journals. Thus, the limited scope of this study may underestimate pharmacist-authored publication rates and trends over time, although the findings were aligned with other similar studies [9,10,11].

There is variation in the way that researchers designate the primary author of a publication (i.e., the primary author can either be the first author or last author). Combining both first and last authors together could have resulted in an overestimation of primary pharmacist authors. Given the lack of change in first- and senior-authored publications over the 20-year period, this limitation was likely minimized.

The study did not delve more deeply into the characteristics of the pharmacist authors, including a breakdown and examination of differences in gender, authors’ institutions and position(s) within the institution, or additional degrees or post-graduate training completed. Given prior literature noting that publication rates were higher in departments offering a PhD program or with federal funding from the National Institutes of Health, these should be focus areas for future research to better understand pharmacist authorship [36].

## 5. Conclusions

Pharmacists are increasingly involved in the conduct and publishing of research across a range of practice areas and topics. This increase in pharmacist-authored publications may be due a variety of reasons, including an increase in pharmacy programs and workforce globally, and greater integration of pharmacists into interprofessional care teams. Despite these positive trends, there remains room for growth in pharmacist research engagement. Use of strategies such as enhanced research training in pharmacy curricula and post-graduate training must be further explored.

## Figures and Tables

**Table 1:** Original research articles in *Journal of the American Medical Association* (*JAMA*) journals and numbers of pharmacist authors.

Journal	Number of Research Articles with Pharmacist Authors from 2000 to 2009 (% of Total)	Total Number of Research Articles from 2000 to 2009	Number of Research Articles with Pharmacist Authors from 2010 to 2019 (% of Total)	Total Number of Research Articles from 2010 to 2019
*JAMA* ^1^	86 (3.5)	2439	114 (4.9)	2338
*JAMA* *Dermatology* ^1^	14 (0.7)	1876	36 (2.3)	1570
*JAMA Internal Medicine*	137 (5.5)	2508	144 (6.4)	2255
*JAMA* *Neurology* ^1^	12 (0.9)	1368	28 (2.2)	1258
*JAMA* *Ophthalmology* ^1^	12 (0.7)	1746	30 (1.6)	1856
*JAMA Otolaryngology—Head and Neck Surgery*	4 (0.2)	1625	8 (0.6)	1327
*JAMA Pediatrics*	21 (1.5)	1397	28 (2.2)	1298
*JAMA Psychiatry*	14 (1.2)	1129	21 (1.8)	1186
*JAMA Surgery* ^1^	15 (0.8)	1771	25 (1.7)	1469

^1^ Statistically significant increases in pharmacist-authored articles in the journal from 2000–2009 to 2010–2019.

**Table 2:** Pharmacist-authored original research articles in *JAMA* journals for each year from 2000 to 2019.

Journal	2000	2001	2002	2003	2004	2005	2006	2007	2008	2009	2010	2011	2012	2013	2014	2015	2016	2017	2018	2019
*JAMA*	4	7	6	17	13	6	10	10	7	6	5	6	9	14	10	16	12	10	15	17
*JAMA Dermatology*	1	2	3	1	1	1	2	0	1	2	2	1	2	3	2	1	4	6	10	5
*JAMA Internal Medicine*	15	13	11	15	11	18	16	14	11	13	21	9	20	12	13	11	15	14	9	20
*JAMA Neurology*	0	1	1	2	0	1	1	3	2	1	1	2	2	1	2	4	2	2	5	7
*JAMA Ophthalmology*	0	1	0	0	2	1	1	1	5	1	4	4	1	3	4	2	2	2	3	5
*JAMA Otolaryngology—Head & Neck Surgery*	0	0	0	0	1	1	0	1	1	0	0	1	1	0	1	1	1	1	0	2
*JAMA Pediatrics*	4	0	2	2	1	1	7	0	1	3	0	1	4	5	3	3	4	4	1	3
*JAMA Psychiatry*	0	1	2	2	2	2	2	0	0	3	2	3	0	2	3	3	1	3	4	0
*JAMA Surgery*	1	3	2	2	2	0	0	2	3	0	2	0	1	3	2	3	6	1	2	5

**Table 3:** Breakdown of pharmacist-authored articles in *JAMA* journals.

Journal	First or Senior Author (% of Total)	Co-Author (% of Total)	First or Senior Author (% of Total)	Co-Author (% of Total)
	2000–2009		2010–2019	
*JAMA*	17 (19.8)	69 (80.2)	21 (18.4)	93 (81.6)
*JAMA Dermatology*	3 (21.4)	11 (78.6)	9 (25.0)	27 (75.0)
*JAMA Internal Medicine*	47 (34.3)	90 (65.7)	60 (41.7)	84 (58.3)
*JAMA Neurology*	0 (0)	12 (100)	5 (16.7)	25 (83.3)
*JAMA Ophthalmology*	6 (50.0)	6 (50.0)	3 (10.0)	27 (90.0)
*JAMA Otolaryngology—Head and Neck Surgery*	1 (25.0)	3 (75.0)	2 (25.0)	6 (75.0)
*JAMA Pediatrics*	9 (42.9)	12 (57.1)	8 (28.9)	20 (71.4)
*JAMA Psychiatry*	5 (35.7)	9 (64.3)	3 (14.3)	18 (85.7)
*JAMA Surgery*	1 (6.7)	14 (93.3)	6 (30.0)	20 (70.0)

## Data Availability

No new data were created or analyzed in this study. Data sharing is not applicable to this article.
